# Leptomeningeal Dissemination in Choroid Plexus Tumors: Magnetic Resonance Imaging Appearance and Risk Factors

**DOI:** 10.3390/children12010082

**Published:** 2025-01-11

**Authors:** Daniel Nunes do Espirito Santo, Monika Warmuth-Metz, Camelia-Maria Monoranu, Martin Hasselblatt, Christian Thomas, Torsten Pietsch, Jürgen Krauß, Tilmann Schweitzer, Brigitte Bison, Matthias Eyrich, Uwe Kordes, Denise Obrecht-Sturm, Mirko Pham, Annika Quenzer

**Affiliations:** 1 Department of Neuroradiology, University Hospital Würzburg, D-97080 Würzburg, Germany; espirito_d@ukw.de (D.N.d.E.S.); warmuth_m@ukw.de (M.W.-M.);; 2 Institute of Pathology, Department of Neuropathology, University of Würzburg, D-97080 Würzburg, Germany; e_monoranu_c@ukw.de; 3 Institute of Neuropathology, University Hospital Münster, D-48149 Münster, Germany; 4 Institute of Pathology, University Hospital Bonn, D-53127 Bonn, Germany; 5 Section of Pediatric Neurosurgery, Department of Neurosurgery, University Hospital Würzburg, D-97080 Würzburg, Germanyschweitzer_t@ukw.de (T.S.); 6 Diagnostic and Interventional Neuroradiology, Faculty of Medicine, University of Augsburg, D-86156 Augsburg, Germany; brigitte.bison@uk-augsburg.de; 7 Neuroradiological Reference Center for the Pediatric Brain Tumor (HIT) Studies of the German Society of Pediatric Oncology and Hematology, Faculty of Medicine, University Augsburg, D-86156 Augsburg, Germany; 8 Department of Pediatric Hematology and Oncology, University Hospital Würzburg, D-97080 Würzburg, Germany; eyrich_m@ukw.de; 9 Pediatric Hematology and Oncology, University Medical Center Hamburg-Eppendorf, D-20246 Hamburg, Germany; u.kordes@uke.de (U.K.);

**Keywords:** choroid plexus tumor, metastasis, MRI, morphology, cysts, imaging

## Abstract

Background: Intracranial choroid plexus tumors (CPT) are rare and primarily affect young children. Leptomeningeal dissemination (LMD) has been reported not only in high-grade choroid plexus carcinoma (CPC) but also in lower histological grades; however, a systematic evaluation of CPT-specific imaging characteristics remains lacking. Methods: We analyzed the imaging characteristics of LMD in a single-center pediatric cohort of 22 CPT patients (thirteen choroid plexus papilloma (CPP), six atypical choroid plexus papilloma (aCPP), three CPC), comparing LMD features with those of the primary tumor. Additionally, we examined the correlation between resection status and LMD development during follow-up. Results: At diagnosis, we observed true LMD in three (two CPCs, one CPP) and pseudo-LMD in one case (CPP). During follow-up, two CPP patients developed cystic LMD, and one aCPP patient developed a solid metastasis. LMD had characteristics of the primary tumor in 3/4 cases. Incomplete resection was associated with a higher risk of LMD (*p* = 0.025). Conclusions: LMD can occur in both high- and lower-grade CPT, presenting at diagnosis as well as in relapsed lower-grade cases. Notable MR-imaging features include pseudo-LMD at diagnosis and cystic LMD in relapsed CPP cases. Incomplete tumor resection may increase the risk of LMD, although further validation is needed.

## 1. Introduction

Choroid plexus tumors (CPT) are rare central nervous system (CNS) tumors arising from choroid plexus epithelial cells. They account for less than 1% of all brain tumors in the overall population but have an increased incidence among infants, representing up to 20% of all tumors in the first year of life [1,2]. According to the 2021 World Health Organization (WHO) classification of CNS tumors, CPT are still classified by histopathological features and divided in three CNS WHO grades: low-grade choroid plexus papilloma (CPP—WHO grade 1), intermediate-grade atypical choroid plexus papilloma (aCPP—WHO grade 2) and high-grade choroid plexus carcinoma (CPC—WHO grade 3) [1]. Histological grading is the most important predictor of overall and event-free survival, with higher-grades being associated with worse clinical outcomes due to increased aggressiveness and associated complication rates [3]. But the intermediate risk of aCPP is restricted to children older than 3 years [4].

Furthermore, DNA methylation profiling of CPTs has revealed three epigenetically distinct and clinically relevant molecular subgroups (“pediatric A”, “pediatric B” and “adult”), demonstrating that some histologically benign CPPs and aCPPs align with high-risk CPCs [5]. Subclass pediatric A (consisting of CPP and aCPP) is of low-risk, while subclass B is of high-risk (consisting of CPP, aCPP and CPC) [6]. Molecular profiling thus provides additional prognostic information that are helpful in guiding treatment decisions.

Several studies indicate that metastases to the subarachnoid space are more common in high-grade CPT compared to lower grade tumors, although these investigations did not include methylation-based risk profiling [7,8]. For instance, CPC present leptomeningeal dissemination (LMD) in up to 21% of all patients at diagnosis, while the incidence in patients diagnosed with aCPP ranges between 5% to 17% [9].

Maximum safe resection is advised for all CPT with careful attention to hemostasis due to an increased risk for bleeding [10,11]. Adjuvant chemotherapy can be utilized to facilitate more successful second-look surgeries [12]. Current guidelines recommend risk-stratified, multimodal therapy. The CPT-SIOP-2000 algorithm incorporated conventional chemotherapy followed by focal radiotherapy in non-metastatic CPC [3]. ACPP patients with residual disease benefit from adjuvant therapy [3]. Additionally, younger age and methylation subgroup “pediatric A” have emerged as favorable prognostic factors in this cohort, suggesting that therapy could potentially be de-escalated [3,13]. For CPP and aCPP with radiologically confirmed metastases, the CPT-SIOP algorithm recommends adjuvant chemotherapy [3].

In this context, prompt identification of LMD at diagnosis and in recurrence has relevant impact on therapeutic decision-making. Although published cohorts have not fully delineated the morphological characteristics of LMD, evidence from case reports suggest that CPTs may present LMD with unique features, such as cystic appearance [14,15,16,17,18,19].

Since the identification of LMD is crucial for therapeutic decision-making, a comprehensive description of its imaging characteristics is essential. We conducted a systematic assessment of LMD in a cohort of pediatric patients with histopathologically confirmed CPT. Our primary objective in this MR-imaging evaluation was to characterize LMD and explore potential correlations with the morphology of the primary tumors. Additionally, we analyzed the data to determine whether the extent of surgical resection influences the incidence of LMD on follow-up imaging.

## 2. Materials and Methods

We performed a retrospective, monocentric analysis of MRI data from patients with histologically confirmed CPT. This study received approval of the local ethical committee (Nr. 2023101002). All pediatric patients underwent resection or biopsy at the Department of Pediatric Neurosurgery at the University Hospital Würzburg, Germany, between 1996 and April 2021. Tumors were histologically assessed and graded on formalin-fixed, paraffin-embedded tissue sections by experienced neuropathologists, following the criteria of the WHO classification. An additional neuropathological reference review was conducted at either the Institute of Neuropathology at the University Hospital Münster, Germany, or the Institute of Neuropathology at the University Hospital Bonn, Germany. The histopathology of all samples was reviewed according to 2021 WHO criteria.

MRIs were evaluated for primary tumor characteristics and appearance of LMD at the time of diagnosis and during serial follow-up after surgical resection. Two board-certified neuroradiologists (A.Q.; M.W.-M.) performed a systematic analysis of the images independently. At diagnosis, the primary tumor location was classified as either supra- or infratentorial. Morphological assessment included evaluating the intensity of contrast enhancement on post-contrast T1-weighted images (T1WI), as well as signal intensity on T1WI and T2-weighted images (T2WI), all rated according to the standardized criteria of the National Reference Center of Neuroradiology for pediatric brain tumor studies [20]. Additionally, the proportion of cystic components, if present, was rated in approximate percentages (<25%, 25–50%; 51–75; >75%). The appearance of blood degradation products or calcifications (summarized as iron deposition) was documented as either present or absent, based on their identification on regular T1WI/T2WI, or, when available, on susceptibility-weighted images (SWI or T2*-weighted images).

LMD at diagnosis or first diagnosed during follow-up examinations, was categorized based on its morphology. The categorization was performed analogously to the modified Chang’s staging system for medulloblastomas, classifying LMD as either intracranial (M2) or spinal (M3), and further specified as having a laminar (a) or nodular (b) configuration [21]. Furthermore, the morphology was described as solid, cystic, or mixed (solid-cystic appearance). The occurrence of diffuse meningeal enhancement at diagnosis, with subsequent spontaneous resolution without any intervention during the follow-up period, was rated as pseudo-LMD. In this context, lesions and meningeal contrast enhancement without spontaneous regression are referred to as “true LMD”.

On early postoperative MRIs, we determined the extent of resection which was categorized as gross-total resection (GTR) or subtotal resection (STR) including debulking. For patients who did not have early postoperative MRI, the resection status was determined based on the surgical report.

Data analysis was performed with SPSS Statistics (IBM Corp. Released 2021. IBM SPSS Statistics for Windows, Version 28.0. Armonk, NY, USA: IBM Corp). Median and range values were provided for continuous variables, while categorical variables were reported as absolute numbers and relative frequencies expressed as percentages. The relationship between the histologically defined CPT groups and the continuous variables (age and tumor volume) was tested using the Mann–Whitney-U or Kruskal–Wallis test. Correlation between CPT groups and categorical variables was evaluated using Fisher’s exact test. These variables included gender, tumor localization, signal-intensity on T1WI and T2WI, presence of cystic components, iron deposition, and contrast enhancement. Additionally, Fisher’s exact test was used to assess potential correlations between the extent of tumor resection (GTR versus STR) and the occurrence of LMD on follow-up examinations. The analysis was conducted exclusively on patients who were not initially metastasized. Despite the exploratory nature of this analysis, *p*-values < 0.05 were considered statistically significant.

## 3. Results

### 3.1. Patients

Thirty patients with histologically confirmed CPTs were identified in our neuropathological archive database. Of these, 22 patients (median age at diagnosis 4.4 years (range 0.2–17.1); male-to-female ratio of 1.4:1; CPP n = 13, aCPP n = 6, and CPC n = 3) had MRI examinations available. However, 4 out of these 22 patients could not be evaluated for the imaging characteristics of the primary tumor because preoperative MRIs were not stored in our institutional medical archive. Consequently, the tumor characteristic analysis was conducted on a smaller group of 18 patients.

For a CPP patient diagnosed before the year 2000 and an aCPP patient from another country who was only operated in Würzburg, the histological diagnosis could not be confirmed by reference pathology. Molecular-genetic results were available in 8/22 patients (6 CPP, 2 aCPP) and the following subtypes were present: “pediatric A” in 3/6 CPP; “pediatric A” in both aCPP; “pediatric B” in 1/6 CPP and “adult” subgroup in 2/6 CPP.

First surgery reached a GTR in 15/22 (10 CPP, 4 aCPP, 1 CPC). Re-resections were performed in 6/7 (2 CPP, 2 aCPP, 2 CPC) and GTR was achieved in 4 (1 CPP, 2 aCPP, 1 CPC).

One CPP patient initially underwent GTR but presented with a local recurrence eight months later, followed by a second GTR. One aCPP patient had an STR, followed shortly after by a GTR, but developed a local recurrence three years later and LMD nearly five years after the initial diagnosis. The cases with recurrences by LMD are later described in detail.

All CPC patients were treated with chemotherapy followed by radiotherapy. The two aCPP patients with STR and two initially metastasized CPP patients received chemotherapy. One CPP patient had four STR within 5 years and was later treated with chemotherapy because of clinical worsening.

Only one of the two CPC patients with LMD at diagnosis died (five years after diagnosis). This patient was tumor-free one year after therapy, but 2.5 years later experienced a local recurrence with extensive spread into the adjacent subdural space. All CPP and aCPP patients were alive, but one aCPP patient was lost to follow-up.

### 3.2. Imaging Characteristics of the Primary Tumor (Table 1)

Of the 18 patients, 12 were confirmed CPP, four aCPP, and two CPC. Both CPCs were located supratentorially, while CPPs were more frequently found infratentorially (8/12). Notably, CPCs exhibited the largest tumor volumes, with a median volume of 174 cm^3^ (range 40–308), whereas CPPs had the smallest, averaging 5 cm^3^ (range 0.4–268; *p* = 0.14). ACPPs had a median tumor volume of 36 cm^3^ (range 25.3–61.5), but the difference was not significant compared to CPPs (*p* = 0.23) and CPCs (*p* = 0.36). Both CPCs showed perifocal edema (median 1.3 cm, range 0.8–1.7), whereas it was present in only 4/12 CPPs (median 0.7 cm, range 0.3–2.6) and in 1/4 aCPPs (1.5 cm). The extent of perifocal edema did not differ significantly among the CPT subgroups (*p* = 0.67).

CPPs were predominantly isointense on T1WI (11/12) and hyperintense on T2WI (8/12). CPCs and aCPPs were more frequently isointense on T2WI (1/1 CPC, 3/4 aCPP). Intratumoral cysts were rather rare among the three subgroups (4/12 CPP, 1/4 aCPP, 1/2 CPC). Both CPCs displayed intratumoral iron depositions, whereas this feature was less present in CPPs (6/12) and aCPP (3/4). Contrast enhancement was strong in all CPCs and 3/4 aCPPs. Even though the majority of CPP presented strong contrast enhancement, in a few cases it was less intense (moderate in 3/12, slight in 1/12). Despite these observations, our analysis did not reveal any significant morphological differences among the three WHO grades.

**Table 1:** Imaging characteristics of the primary tumors.

	CPP (n = 12)	aCPP (n = 4)	CPC (n = 2)	*p* Value
age at diagnosis median and range in years	8.3 (0.2–17.1)	1.3 (0.3–4.2)	8.2 (0.7–15.6)	0.127
gender				0.029
• male	10 (83.3%)	2 (50%)	-	
• female	2 (16.7%)	2 (50%)	2 (100%)	
tumor localization				0.164
• IT	8 (66.7%)	1 (25%)	-	
• ST	4 (33.3%)	3 (75%)	2 (100%)	
tumor volume median (range) in cm^3^	5.2 (0.45–268.1)	35.8 (9.5–61.5)	173.9 (39.9–308)	0.196
perifocal edema				0.349
• present	4 (33.3%)	1 (25%)	2 (100%)	
• absent	8 (66.7%)	3 (75%)	-	
T1 signal intensity				1
• isointense	11 (91.7%)	4 (100%)	2 (100%)	
• hyperintense	1 (8.3%)	-	-	
• hypointense	-	-	-	
T2 signal intensity				1
• isointense	4 (33.3%)	1 (25%)	1 (50%)	
• hypointense	-	-	-	
• hyperintense	8 (66.7%)	3 (75%)	1 (50%)	
cystic components				1
• absent	8 (66.7%)	3 (75%)	1 (50%)	
• <25%	2 (16.7%)	-	-	
• 26–50%	1 (8.3%)	1 (25%)	1 (50%)	
• 51–75%	1 (8.3%)	-	-	
• >75%	-	-	-	
iron deposition				0.465
• present	6 (50%)	3 (75%)	2 (100%)	
• absent	6 (50%)	1 (25%)	0	
contrast enhancement				1
• slight	1 (8.3%)	0	0	
• moderate	3 (25%)	1 (25%)	0	
• strong	8 (66.7%)	3 (75%)	2 (100%)	

Table 1 presents the morphological analysis of the primary tumor, which was performed in 18 out of 22 patients since MRI at diagnosis was not available for four patients. Absolute and relative frequencies are given. Abbreviations: ST, supratentorial; IT, infratentorial; CPP, choroid plexus papilloma; aCPP, atypical choroid plexus papilloma; CPC, choroid plexus carcinoma.

### 3.3. Leptomeningeal Dissemination (Table 2)

#### 3.3.1. At Diagnosis

**Figure 1:**
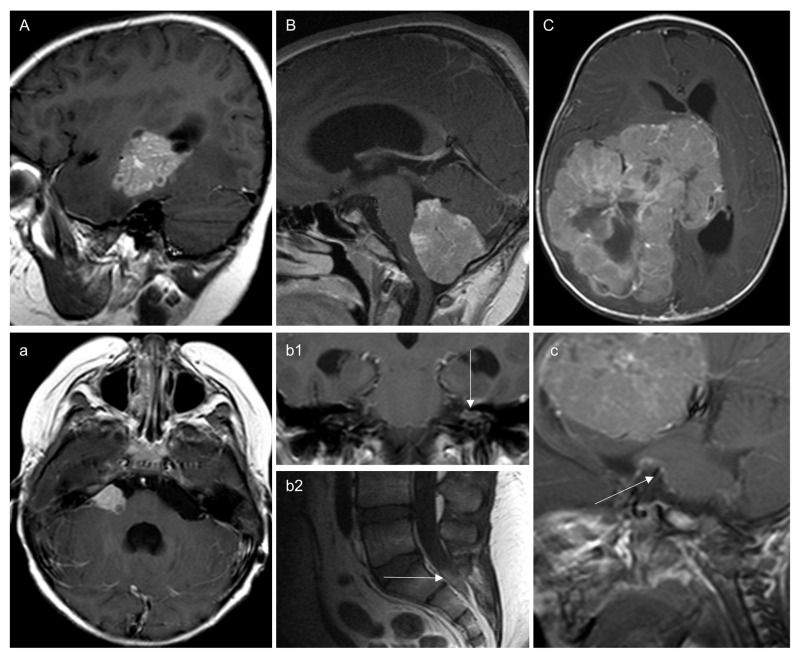
Figure presents all cases with true leptomeningeal dissemination (LMD) at diagnosis. (**A**) Case of a supratentorial choroid plexus carcinoma with simultaneous LMD in the right cerebellopontine angle (**a**), which, according to imaging characteristics, is very similar to the tumor and exhibits small cystic components. (**B**) Case of a purely solid plexus papilloma with also solid LMD in the left internal auditory meatus (arrow in **b1**) as well at the level S1/2 (arrow in **b2**). (**C**) Case of a large supratentorial choroid plexus carcinoma with laminar LMD along the anterior brainstem (arrow in **c**).

At diagnosis, 3/22 patients presented true LMD (one CPP and two CPCs; no molecular genetic information available; Figure 1). One CPC patient presented M2a, while the second one exhibited M2b with mixed solid-cystic features and M3b. The CPC patient with M2b and M3b had no pre- but an early postoperative MRI following STR, but the imaging features of the residual tumor were comparable to that of the LMD.

**Table 2:** Incidence of leptomeningeal dissemination at diagnosis and on follow-up examinations per histological subgroup.

	CPP (n = 13)	aCPP (n = 6)	CPC (n = 3)
resection status			
• GTR	10 *	4	1
• STR	3	2	2
LMD at diagnosis	n = 2	n = 0	n = 2
classification			
• M2a	1	-	1
• M2b + M3b	1	-	1
morphology			
• solid	1	-	1
• mixed	-	-	1
• pseudo LMD	1	-	
LMD on follow-up	n = 2	n = 1	n = 0
classification			
• M2b	2	1	-
morphology			
• solid	-	1	-
• cystic	2	-	-

Abbreviations: CPP, choroid plexus papilloma; aCPP, atypical choroid plexus papilloma; CPC, choroid plexus carcinoma; GTR, gross total resection; LMD, leptomeningeal dissemination; n, number; M2a, intracranial laminar LMD; M2b, intracranial nodular LMD; M3b, spinal nodular LMD; STR, subtotal resection. * Including one patient where early postoperative MRI was unavailable, but surgery report assessed total resection and on follow-up no residual tumor was present.

One CPP patient presented with solid M2b and M3b. In that case, the contrast enhancement pattern and the absence of cystic portions were comparable to the primary tumor.

**Figure 2:**
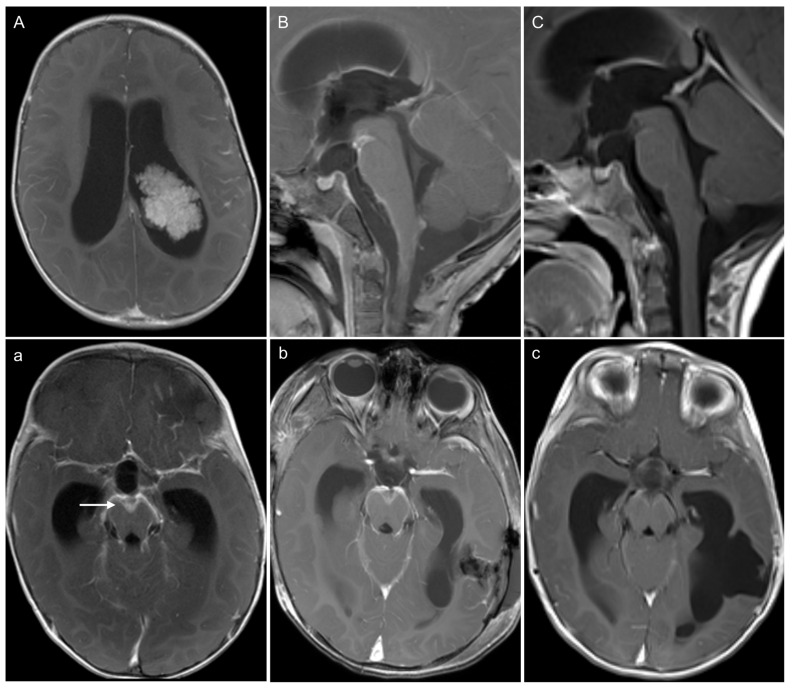
A 1-year-old male CPP patient with pseudo leptomeningeal dissemination at diagnosis. Contrast-enhanced T1-wheigted images show the course from presurgery to pre-chemotherapy in sagittal (**A**–**C**) and transverse planes (**a**–**c**). (**A**,**a**) Contrast enhanced T1-wheighted image shows the primary tumor and the diffuse leptomeningeal contrast enhancement at the skull base, especially in the interpeduncular cistern as demonstrated in (arrow in **a**). (**B**,**b**) The diffuse contrast enhancement was still detectable immediately after surgery. (**C**,**c**) However, the contrast enhancement had regressed in an MRI performed 4 weeks later before the start of chemotherapy.

Additionally, we observed one case of CPP (“pediatric B” subtype) with diffuse laminar leptomeningeal enhancement at diagnosis, which was initially rated as M2a but then showed spontaneous remission pre-therapy on the follow-up MRI examination without any interventions, being categorized as pseudo-LMD (Figure 2).

#### 3.3.2. On Follow-Up

**Figure 3:**
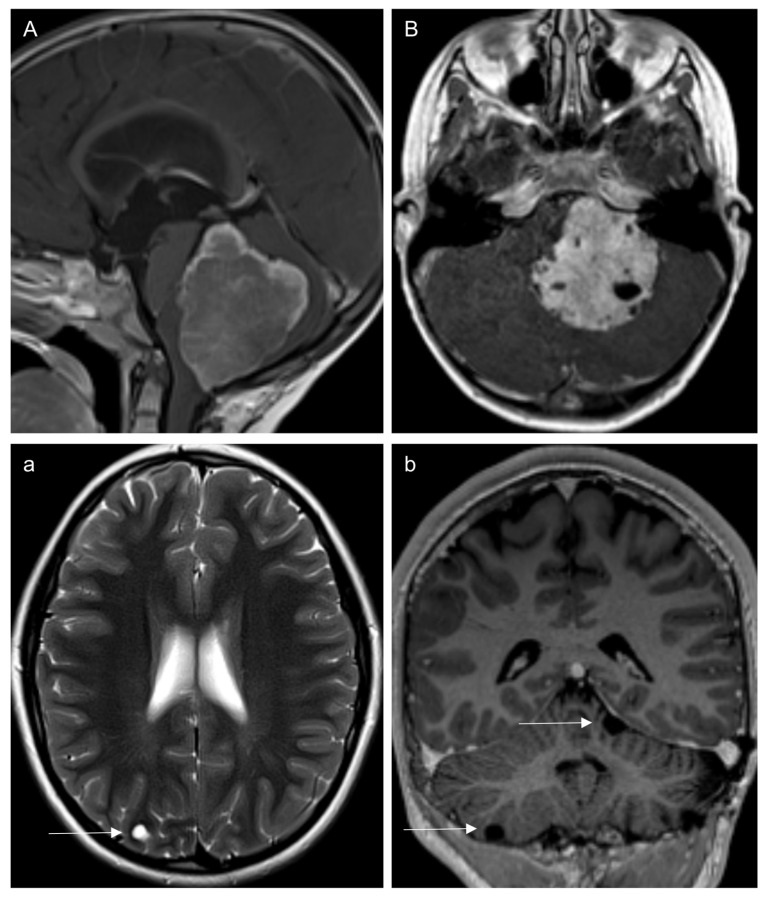
This Figure shows the two CPP patients, who presented with new cystic leptomeningeal lesions during follow-up after incomplete primary tumor resection. (**A**) A 3 years old male patient with CPP. The primary tumor had no cystic components. Three months after surgery, first cystic leptomeningeal lesions were noticed (arrow in **a**). (**B**) A 6 years old male CPP patient initially presented intratumoral cysts. Cystic leptomeningeal lesions without contrast enhancement appeared four years after diagnosis (arrows in **b**).

During the follow-up period, three patients developed recurrence by LMD (two infratentorial CPP, one supratentorial aCPP). The median time to LMD was 3.9 years (range 0.3–4.6). At primary diagnosis, both CPP patients underwent STR, with one achieving a near-complete STR. Both patients presented postoperative blood degradation products at the primary tumor site. Neither of the CPP patients received radio- or chemotherapy following primary tumor resection, and LMD relapse occurred at 3 and 48 months post-diagnosis. Both individuals presented with exclusively cystic LMD, as shown in Figure 3. On baseline examinations, only one of these CPPs cases exhibited a primary tumor with cystic components.

The CPP (“pediatric A” subtype) patient with near-complete STR developed cystic LMD three months after surgery. The local tumor showed slow progression over a period of 22 months, after which it was completely resected. Over a 6-year follow-up period, this patient also showed an increase in the number of cystic arachnoidal lesions within both the posterior fossa and supratentorial compartment; however, these were all very small and not space-occupying. The patient was alive at a 7-year follow-up.

The second CPP (“adult” subtype) patient, who underwent STR, had a re-resection that again achieved STR. Over the next 17 months, the residual tumor showed gradual progression, leading to another STR. Four years after the initial diagnosis, new and enlarging subarachnoid cysts appeared in the posterior fossa, although these also remained non-space-occupying. At that time, the residual primary tumor displayed further progression. During the follow-up, one significantly progressive intratumoral cyst required resection five years post-diagnosis. Due to tumor-associated low-frequency hearing loss nearly six years from diagnosis, the patient began chemotherapy according to the CPT-SIOP protocol. The patient was alive after an 8-year follow-up.

The aCPP patient (male, five years old) initially achieved STR and later reached GTR status following a second-look surgery two months later. In that case, no molecular genetic information was available. Postoperative chemotherapy was administered for consolidation. Three years after the initial diagnosis, a locally recurrent tumor developed and was completely resected. Fifty-four months from the initial diagnosis and 13 months after the resection of the local recurrence, the patient developed solid M2b, without cystic components, in the infundibular recess. At that time, no local residual primary tumor was present. Since no initial MRI was available for this patient, a comparison with the morphology of the primary tumor was not possible. The M2b showed slow progression over a five-year period, and no further interventions were undertaken. Afterwards that patient was lost for a further follow-up.

In summary, 2/3 LMD cases at diagnosis and the single evaluable LMD case at relapse presented imaging features similar to the primary tumor (total 3/4). Progressive diseases through LMD occurred exclusively in patients who initially underwent STR (Table 3). STR of primarily non-metastatic CPT was significantly associated with LMD occurrence during follow-up (*p* = 0.025). However, within each histological subgroup, this association was not observed (CPP *p* = 0.11; aCPP *p* = 0.33; n = 1 CPC, *p* not evaluable).

## 4. Discussion

This study cohort summarizes the unique imaging characteristics of LMD in CPT. Particularly noteworthy is the pre-therapeutic diffuse leptomeningeal enhancement, which we interpreted as pseudo-LMD, and the cystic appearance of LMD in CPP.

The identification of relapse in brain tumors may be influenced by the contrast enhancement pattern [22]. Contrast enhanced MRI is standard on follow-up and contrast enhancing lesions are more likely to be evaluated as recurrence. However, it is known from other brain tumor entities that the imaging characteristics of metastases not necessarily reflect those of the primary tumor [23]. Hence, we assessed both the characteristics of the CPT metastases and those of the primary tumor.

Despite variations in histopathological grade, our cohort of CPTs predominantly exhibited isointensity on T1WI, hyper- or isointensity on T2WI, and strong contrast enhancement. Lower degrees of contrast enhancement were exclusively observed in the lower grade CPT. Cystic intratumoral lesions were most often absent (8/12 CPP, 3/4 aCPP, 1/2 CPC). While 3/4 aCPPs and all CPCs exhibited iron depositions within the tumor, this was the case in fewer than 50% of CPPs. However, due to small patient numbers these morphological differences did not reach statistical significance.

Previous research in slightly larger cohorts has shown significant correlations between the morphological characteristics and distinct CPT subgroups. Research by Chen and coworkers evaluated differences in imaging characteristics between CPP and aCPP [24]. They reported higher median tumor volumes, lower signal intensity in T2WI, and an increased incidence of intratumoral cysts in aCPPs (n = 9) compared to CPPs (n = 12). The disparity between the results of Chen et al. and ours could be explained by a discrepancy in the aCPP cohort size (n = 9 vs. n = 4).

Significant differences between the three histological groups were also found in the study by Lin et al. [25]. CPCs were shown to have significantly larger tumor diameters and to present with perifocal edema, whereas neither CPP nor aCPP cases presented edema. Additionally, the authors reported a purely cystic appearance in 25% of CPPs. In our cohort, CPCs were also considerably larger than CPPs, though this difference was not statistically significant, possibly due to small group sizes and to differing measurement methods: Lin et al. measured the longest diameter, while we measured tumor dimensions in all three planes to calculate volumes. Furthermore, none of the CPP cases in our cohort presented without solid tumor components. Since we only found one more case report in the literature describing a purely cystic CPP [26], this particular variant appears to be quite rare.

Within our cohort, two out of three CPC patients demonstrated LMD at diagnosis, while only one CPP and no aCPP had LMD. The CPT-SIOP-2000 study reported LMD at diagnosis in 17% of aCPP [9]. Given our small sample size and limitation to a single-center experience, the incidence of LMD is likely underestimated in the aCPP group.

One CPP patient presented a solid contrast-enhancing M2b at diagnosis, which is a not unusual presentation in benign CPT [19,27]. That case of M2b showed no imaging differences to the primary tumor. In the CPC patients, LMD appeared at diagnosis laminar in one case and mixed solid-cystic in the second case, with the latter resembling the features of the primary tumor.

One patient with CPP presented at diagnosis with pathological diffuse laminar leptomeningeal enhancement, which was initially rated as laminar M2a. But it then regressed spontaneously before the initiation of chemotherapy, leading to its categorization as pseudo-LMD. Similar phenomena are highlighted in patients with CPP and aCPP in several case reports [28,29,30,31]. These patients also showed spontaneous regression of the diffuse laminar enhancement following tumor resection, without any additional therapeutic intervention. It is hypothesized that this phenomenon might be a result of abnormal arachnoidal vessel permeability induced by hormonal factors [28,31,32]. Heese et al. raised the hypothesis that there might be true arachnoidal infiltration of a thin layer of tumor cells dependent upon critical growth factors in the CSF secreted by the tumor, which regresses after complete tumor resection and the subsequent loss of triggering factors [30]. Intraoperatively, Heese and colleagues found abnormal arachnoidal appearance with a thin gelatinous layer but a sample for further examination was not taken. Spontaneous regression of leptomeningeal enhancement along the brainstem has also been reported in glioblastomas [33]. The authors suggested that glioblastomas, due to the presence of intratumoral arteriovenous shunts, perfuse deep veins through the shunts, leading to enhancement of the brainstem. Since CPTs can acquire unusual histological features, such as an angioma-like increase in blood vessels [1], this hypothesis cannot be excluded. Therefore, spontaneously remitting leptomeningeal enhancement in lower grade CPT points to a potentially underexplored aspect of LMD that warrants further investigation. In such instances, patients may benefit from a cautious therapeutic approach. It could be advantageous to delay chemotherapy/radiotherapy until a short-term follow-up examination post-surgery was conducted to confirm this pattern, potentially helping to avoid unnecessary treatment.

Resection is the mainstay in primary treatment of CPT. Several studies on pediatric, adult and mixed pediatric and adult cohorts showed that GTR was associated with higher survival rates [34,35,36,37] and lower recurrence rates [35]. The CPT-SIOP-2000 study did not show a significant impact of residual tumor on event-free or overall survival [3]. In their CPC group, GTR vs. STR vs. biopsy did not influence overall survival. The authors concluded that this unexpected finding was possibly caused by optimized post-surgical treatment regimes.

In this work, LMD relapse was exclusively observed in lower grade CPT patients after initial STR (two CPP and one aCPP). This finding should be interpreted with caution since our results might be influenced by the small sample size and were not significant for the histological subtypes. However, further validation of this observation is necessary.

In our single aCPP patient with relapse by solid LMD we could not compare to the primary tumor, because the initial MRI was lacking. Among our CPP patients with cystic LMD, only one had a primary tumor with cystic components, suggesting that primary tumor morphology may not influence the appearance of LMD. Furthermore, we found no reports of purely cystic LMD in patients with high-grade CPT, leading us to conclude that the development of cystic LMD may be a distinctive feature of lower-grade CPTs. Cases in a recently published review article were consistent with the cystic LMD pattern observed in our cohort [14]. In that review, cystic LMD was mainly noted in CPP patients, with one case in an aCPP patient [18]. However, most patients in the review presented with cystic LMD at diagnosis, while only one case presented cystic LMD eight years postoperatively [19]. In contrast, we detected cystic LMD only in CPP patients and during follow-up. In one case, however, the first cyst was identified just three months post-surgery, which was close to the time of diagnosis.

As in some of the collected cases by Johnson et al. [14], our patients showed a slow progression of the leptomeningeal cystic lesions as well. Both the small size and the number of cystic lesions typically argue against immediate resection. Within the reviewed cases, treatment regimens with chemotherapy and radio-chemotherapy showed good clinical response or stabilization in single cystic lesions [18,19]. A biopsy from a cystic leptomeningeal lesion revealed predominantly normal choroid plexus tissue. However, focal areas of slightly increased cellularity were also observed, a characteristic feature of CPP [19]. This observation supports our assumption that the cystic lesions detected in our study group represent true LMD, despite the lack of histological confirmation.

The exact pathophysiology of cystic LMD remains unclear. Based on histological findings, Johnson et al. [14] postulated that CPTs may release ‘unhinged’ arachnoid cap cells with high secretory activity, potentially leading to aberrant secretion responsible for the formation of these cystic lesions. Postoperative subarachnoid adhesions have been proposed as another possible cause; however, this hypothesis does not explain the occurrence of supratentorial cystic lesions in a case of primarily infratentorial CPP. Furthermore, if subarachnoid adhesions were the primary mechanism, one would expect cystic lesions in other tumor entities as well, as for example perioperative bleeding is not uncommon.

Our study is limited by the small sample size and the occasional absence of initial MRI examinations. Furthermore, we are unable to provide full genetic information of the primary tumors and histological findings for the LMDs. Comprehensive future research is essential to fully determine the nature and extent of the relationship between resection status, histology and genetic subclass, and the incidence of LMD at relapse, with the ultimate goal to refine treatment strategies for CPT patients. In this context, radiologists should be able to identify the specific morphology of LMD in CPTs.

## Figures and Tables

**Table 3:** Correlation between the resection status of primary non-metastatic CPTs and the later occurrence of LMD.

		LMD	No LMD
CPP	total n = 10, *p* = 0.11	2 (20%)	8 (80%)
	GTR	0	6 (75%)
	STR	2 (100%)	2 (25%)
aCPP	total n = 6, *p* = 0.33	1 (16.7%)	5 (83.3%)
	GTR	0	4 (80%)
	STR	1 (100%)	1 (20%)
CPC	total n = 1	-	1 (100%)
	GTR	-	1 (100%)
	STR	-	-

Abbreviations: aCPP, atypical choroid plexus papilloma; CPP, choroid plexus papilloma; CPC, choroid plexus carcinoma; GTR, gross total resection; LMD, leptomeningeal dissemination; n, number; STR, subtotal resection.

## Data Availability

The data presented in this study are available upon reasonable request from the corresponding author.
